# The PATHS survey: a participatory-informed, multilingual instrument for assessing tobacco and nicotine-related knowledge, attitudes, and behaviours among people with a migration background in Switzerland

**DOI:** 10.3389/fpubh.2026.1872320

**Published:** 2026-08-26

**Authors:** Kris Schürch, Tayisiya Krasnova, Nathalia Gonzalez-Jaramillo, Emilia B. Nordmeyer, Luciano Ruggia, Annika Frahsa

**Affiliations:** 1 Swiss Association for Tobacco Control (AT Switzerland), Bern, Switzerland; 2 Graduate School for Health Sciences, University of Bern, Bern, Switzerland; 3 Institute for Social and Preventive Medicine, Department of Preclinical Medicine, Faculty of Medicine, University of Bern, Bern, Switzerland

**Keywords:** Switzerland, participatory-informed, multilingual survey design, tobacco prevention, migration background

## Abstract

**Introduction:**

Research on tobacco and nicotine use among people with migration backgrounds is characterised by methodological limitations, including a predominant focus on behavioural outcomes, limited integration of knowledge and attitudes, and a lack of participatory approaches. Standard population surveys further restrict linguistic accessibility and often fail to capture the complexity of migration-related experiences, thereby limiting the interpretability of observed health inequalities. We thus aimed to develop a multilingual, participatory survey instrument assessing knowledge, attitudes, and behaviours (KAB) related to tobacco and nicotine use among people with migration backgrounds in Switzerland.

**Methods:**

The Perspectives and Attitudes Towards Health and Smoking (PATHS) survey was developed following the WHO framework for knowledge, attitude and practice surveys, informed by Cognitive Aspects of Survey Methodology (CASM) and KAB frameworks. We applied a participatory-informed approach with iterative community consultations across all development phases, including evidence mapping, survey design, translation, and pilot testing. We adapted and extended validated items from international and national surveys (e.g., WHO GATS, Swiss Health Survey). We translated the instrument in collaboration with community partners and iteratively refined through partner feedback.

**Results:**

The final instrument comprises 136 items covering the different KAB domains, sociodemographic characteristics, and multidimensional migration-related variables. It operationalises migration background beyond categorical indicators, incorporating factors such as migration history, legal status, language use, and reasons for migration. Community input informed content expansion, wording simplification, and the inclusion of visual aids. Pilot testing indicated good feasibility (10–15 min completion time; ~80% completion rate). A community-based dissemination strategy was established, supported by multilingual materials and a network of 53 partner organisations.

**Conclusion:**

The PATHS survey demonstrates how participatory, multilingual approaches can enhance the inclusiveness and explanatory power of survey-based research. This methodology provides a transferable framework for studying health behaviours in diverse populations.

## 1 Introduction

Research on tobacco and nicotine products among people with migration backgrounds is characterised by methodological imbalances that limit its explanatory potential and practical usefulness (1). Existing studies often focus primarily on behavioural outcomes of tobacco smoking, such as current smoking status or product use, while giving less attention to knowledge, and attitudes towards tobacco and nicotine products (1–3). In addition, migration background is frequently operationalised through binary or static categories, limiting the ability to capture heterogeneity in migration-related experiences, language use, social contexts and exposure to different tobacco environments (4–9). Participatory approaches that involve people with migration backgrounds or community actors in the design of research instruments remain underused, although such approaches may improve the relevance, accessibility and equity of public health research (1, 10–12).

These limitations are particularly relevant in Switzerland. The country has a persistently high tobacco smoking prevalence, a large resident population with a migration background (≥40%), combined with comparatively weak national tobacco control policies and evidence gaps on tobacco and nicotine use (12–16). Analyses of nationally representative Swiss Health Survey 2022 data showed that among Swiss residents aged 15 years and above, people with migration backgrounds had elevated odds of current tobacco smoking in comparison to those without a migration background, with particularly elevated odds among younger second-migration generation respondents and respondents with lower educational attainment (see Table 1 for definition of people with migration background) (17). These findings demonstrate the value of national surveillance data for identifying tobacco-related inequalities. At the same time, they also point to the need for complementary instruments capable of generating more explanatory evidence on the knowledge, attitudes, and contextual factors that may shape tobacco and nicotine product use among people with migration backgrounds.

**Table 1:** Definitions of terms (Bern, Switzerland, 2026).

Term	Definition
People with migration background	We use the term *people with migration background* to refer to individuals who have migrated themselves or whose parents have migrated, encompassing both first-generation migrants and subsequent generations. This definition aligns with international migration literature, including distinctions based on the International Organization for Migration definition for first-generation migrants and the classification of second-generation individuals as those with at least one parent born abroad (66–68). It is also consistent with terminology used by the European Commission, Dutch policymaking, and the Swiss Federal Statistical Office (14, 69, 70). This broad terminology captures diverse migration histories, including individuals who may not have migrated themselves, but whose lives are shaped by intergenerational mobility. We deliberately adopt this term to move beyond the narrower and potentially stigmatising label of “migrant,” acknowledging critiques that such labels can problematise mobility and essentialise heterogeneous populations (55). Accordingly, the term *people with a migration background* reflects a more inclusive and analytically flexible perspective that recognises migration as a heterogeneous, relational, and context-dependent set of experiences rather than a singular or static category.
Knowledge	*Knowledge* (K) embodies “all information that a person possesses or accrues related to a particular field of study” (24). For smoking, this can be in regard to knowledge of harmful effects, or perception of health effects of smoking (e.g., “smoking is harmful,” “smoking can cause heart diseases”) (54).
Attitudes	*Attitudes* (A) refer to the subjective “sum or aggregate of all feelings and dispositions toward a particular concept, idea or action” (71). Attitudes can be a belief or idea associated with a particular object, can represent the individual’s evaluation and emotion associated with the object, or attitudes can represent the predisposition of action towards the object (24). Smoking related attitudes include “smoking is pleasurable,” “smoking relaxes me,” “smoking helps me lose weight” (23).
Behaviours	*Behaviours* (B) are understood as an observable action. In other words, the way a person, or group of persons act in certain conditions (24). Smoking related behaviours include “tobacco use among Somali adults in Minnesota,” “hookah smoking amongst Eritrean adults.”

The Swiss Health Survey provides an important source of nationally representative data and uses a clear classification of migration background based on country of birth (17, 18). However, like many population-based surveillance instruments, it is primarily designed to monitor health behaviours and population-level indicators. It therefore provides more limited insight into respondents’ knowledge of tobacco and nicotine-product related harms, attitudes towards specific products, product-specific risk perceptions, and the migration-related contexts in which tobacco and nicotine product use occurs (17).

Furthermore, the Swiss Health Survey is administered in the three main Swiss national languages (German, French, and Italian), which may limit accessibility for individuals with limited proficiency in these languages (18). This can affect whose perspectives are represented in survey data and may reduce the visibility of groups with different linguistic repertoires, migration histories, or experiences of integration (19, 20).

**Figure 1:**
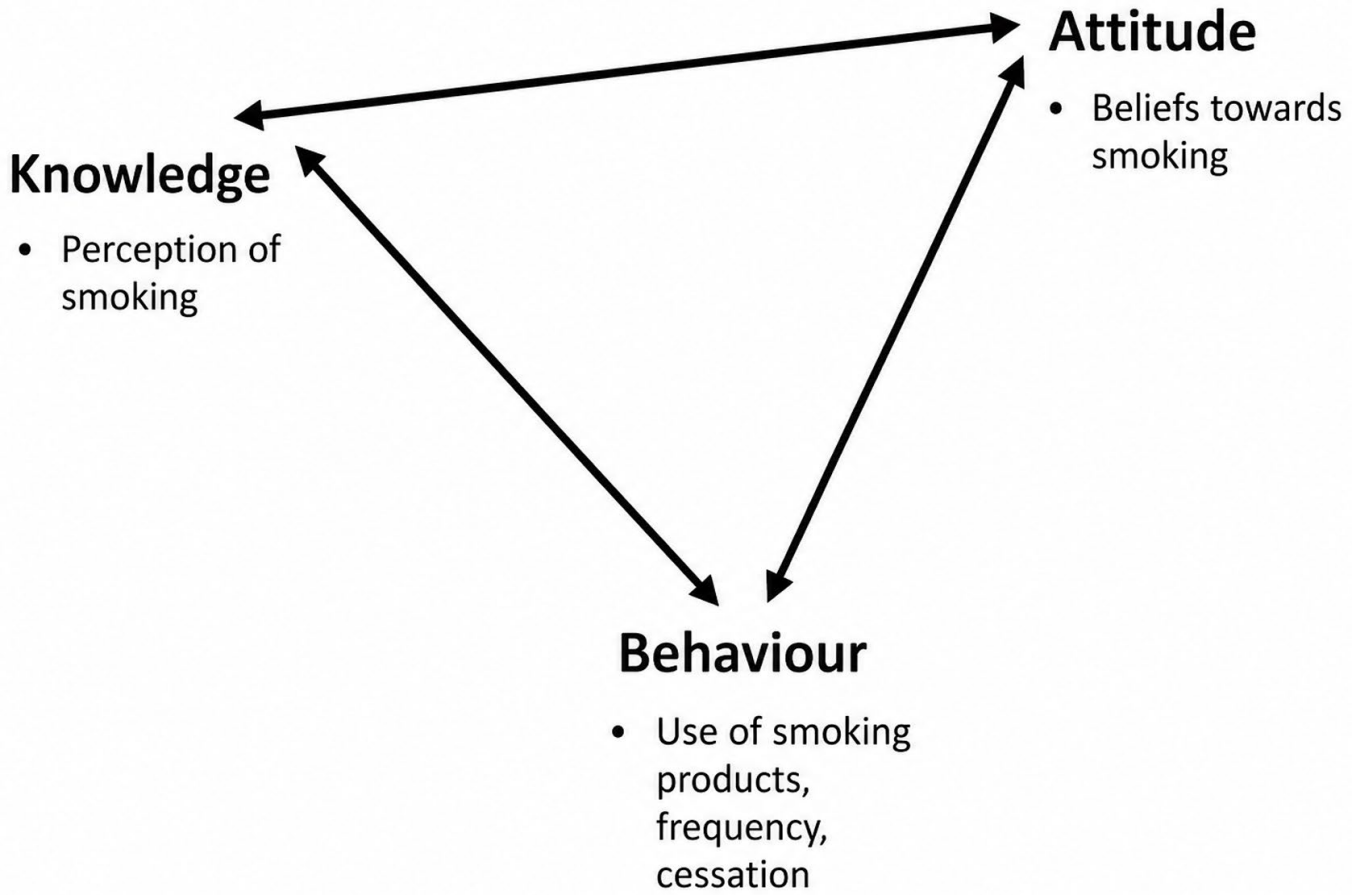
Simplified model of knowledge-attitude-behaviour consistency (Chaffee & Roser) (Bern, Switzerland, 2026).

A knowledge, attitudes and behaviours (KAB) framework offers a useful conceptual approach for addressing these gaps. Tobacco and nicotine product use among people with migration backgrounds cannot be understood through behavioural indicators alone. Product use may be shaped by access to language-appropriate health information, previous exposure to different tobacco environments, social norms within families or communities, experiences of integration or marginalisation, and perceptions of the harms, addictiveness or social acceptability of different products (6, 21–23). A KAB framework allows behaviours to be interpreted in relation to what people know, how they evaluate tobacco and nicotine products, and how these dimensions interact with social and migration-related contexts (see Table 1 for definitions of KAB) (24–26). In this sense, KAB does not assume a linear relationship between knowledge, attitudes and behaviours, but provides a structure for examining their dynamic and context-dependent interplay (see Figure 1 of simplified model by Chaffee & Roser on KAB consistency).

In addition to deciding what a survey should measure, instrument development must also consider how respondents understand, interpret and answer survey questions. Cognitive Aspects of Survey Methodology (CASM) conceptualises survey answering as a process involving comprehension, retrieval of relevant information, judgement and response mapping (27). This is particularly important in multilingual and migration-sensitive survey research, where question wording, translation choices, cultural interpretation and response options may affect how respondents understand and answer items. CASM therefore provides a methodological rationale for iterative refinement, linguistic adaptation, pilot testing and the use of participatory feedback to improve comprehensibility and accessibility.

From a participant-centered perspective, research instruments developed without input from the populations they aim to study may reproduce institutional assumptions about relevance, accessibility and interpretation. Missing input can limit the consideration of linguistic and cultural diversity, particularly when affected populations are included only as respondents rather than as contributors to survey design (10, 11). Participatory approaches can improve the reach, relevance, and equity of public health research and interventions, especially when working with populations that may be underrepresented in standard health surveys (10–12, 28, 29).

Recent global and European public health frameworks have begun to emphasise that evidence gaps regarding migrant and marginalised population health are not merely a consequence of limited data availability but are linked to research designs that insufficiently account for linguistic diversity, lived experience, and contextual heterogeneity. The World Health Organization’s (WHO) global research agenda on health, migration, and displacement from 2023, the WHO’s investment case on health and migration from 2024, and a 2025 editorial by The Lancet titled “*The uncertain future of migrant and refugee health*” converge to highlight the need for people-centred and inclusive research to help guide towards migrant sensitive health-systems (30–32). Likewise, the European Commission’s Safe Hearts Plan approved in December 2025 emphasises people-centred non-communicable disease prevention strategies that engage affected populations in the design of health actions (33). These global agendas signal a growing consensus that participatory approaches are not solely ethical considerations but essential methodological foundations to producing meaningful and useful evidence in diverse populations.

We therefore aimed to develop a participatory-informed, multilingual survey instrument for assessing tobacco and nicotine product-related knowledge, attitudes and behaviours among people with migration backgrounds in Switzerland, which would complement existing population-based surveillance by providing a more linguistically accessible, migration-sensitive and KAB-oriented instrument for generating explanatory evidence.

## 2 Methodology

### 2.1 Conceptual and methodological orientation

The survey project “Perspectives and Attitudes Towards Health and Smoking (PATHS)” was developed using a participatory-informed, co-creation-oriented approach to instrument development. Building on the conceptual rationale outlined in the Introduction section, KAB and CASM served distinct but complementary roles in the development of the survey. The WHO guide to developing Knowledge, Attitude and Practices Surveys informed what the instrument measured by structuring the questionnaire around knowledge, attitudinal and behavioural domains related to tobacco and nicotine products (26). CASM informed how survey items were designed and refined by guiding attention to question comprehension, information retrieval, judgement formation and response mapping, particularly in the context of multilingual survey administration (27).

Drawing on participatory and co-creation principles, the participatory-informed approach integrated experiential, practice-based, and contextual knowledge alongside academic expertise (28, 29, 34–38). Rather than treating individuals with migration backgrounds solely as respondents, the development process incorporated iterative feedback from community actors. This input contributed to the relevance, accessibility, comprehensibility and cultural and linguistic appropriateness of the instrument. The process was not designed as a full community-based participatory research project with shared decision-making and governance across all phases, we instead had varying levels of participation across the process rather than a single fixed level (10, 39–41). Drawing on the participatory research choice-points model, the level of involvement differed according to the development phase and task: community and practice actors contributed through consultation, iterative involvement and collaborative refinement, while the research team retained responsibility for final methodological and feasibility decisions. The approach is therefore described as participatory-informed and co-creation-oriented rather than as full community-based participatory research, which would imply equitable shared governance across all phases of the research process (29, 35, 38, 42).

Similarly, the instrument was not designed as a full psychometric validation study or as a formal implementation of the TRAPD (Translation, Review, Adjudication, Pretesting, Documentation) or COSMIN (Consensus-based Standards for the selection of health Measurement Instruments) frameworks (43). However, the development process incorporated several principles emphasised in established methodological standards for instrument development and cross-cultural survey adaptation. In relation to COSMIN, this included attention to content validity, relevance and comprehensibility. In relation to TRAPD, this included translation, review, adjudication, pretesting and documentation as reference points for multilingual survey development (43, 44).

### 2.2 Overview of PATHS project development

**Figure 2:**
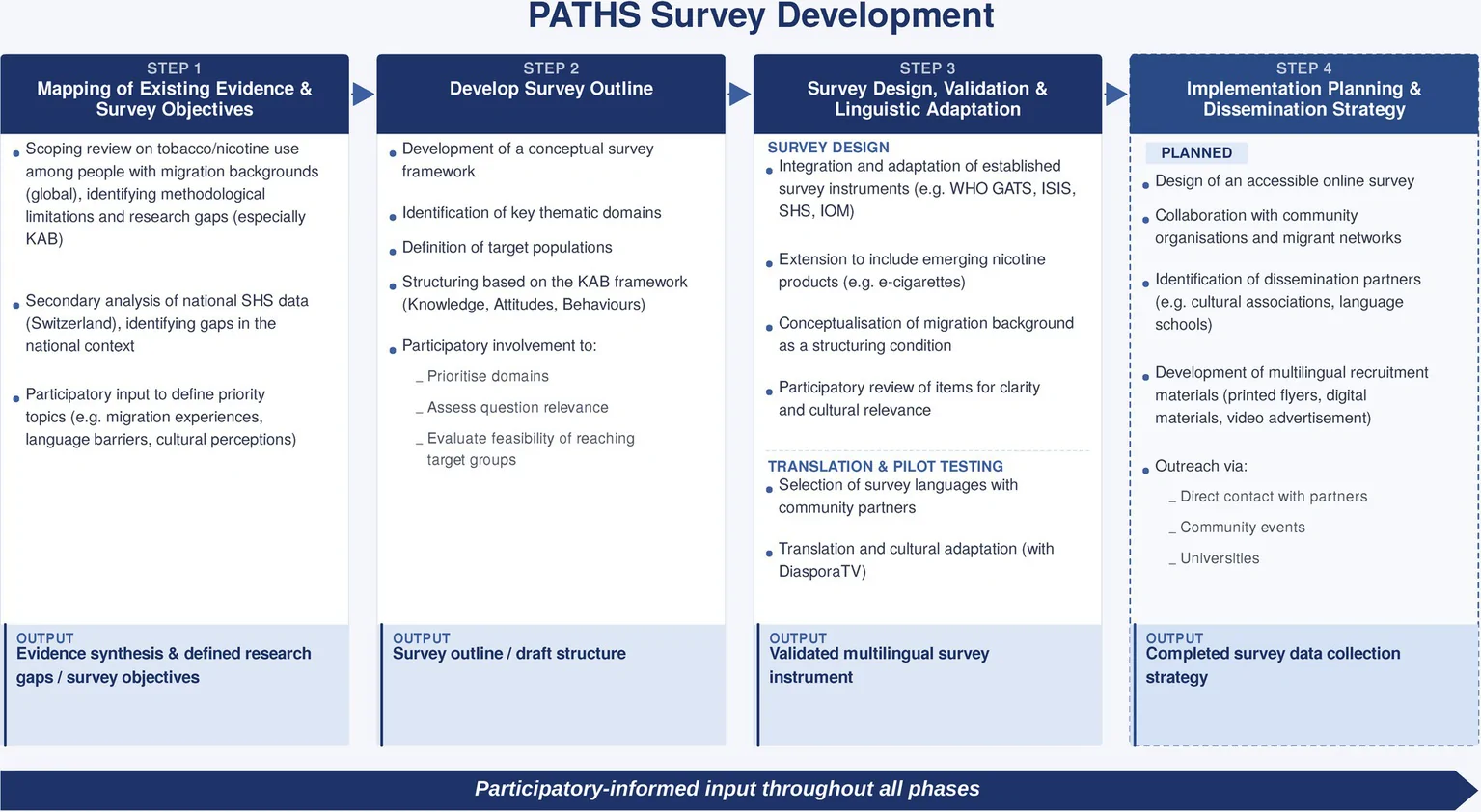
Four phases of PATHS survey development (Bern, Switzerland, 2026).

The PATHS survey was developed in four steps, adapted from the WHO guide to developing knowledge, attitude and practice surveys: (1) mapping existing evidence and defining survey objectives; (2) developing the survey outline; (3) survey design, item adaptation, translation, linguistic refinement and pilot testing; and (4) implementation planning and dissemination strategy. Figure 2 provides an overview of this development process. Participatory input was integrated across all four steps, although the level and form of engagement varied according to the task, actor group and development phase. Table 2 summarises the actor groups involved, input formats, main contributions and concrete adaptations retained in the final PATHS instrument.

**Table 2:** Overview of PATHS participatory-informed input (Bern, Switzerland, 2026).

PATHS development phase	Actor groups involved	Input formats	Main contributions	Concrete adaptations retained in the final instrument
Step 1: Mapping existing evidence and defining survey objectives	Research team; public health practitioners; migrant community actors	Evidence mapping; expert exchanges; workshop-based and informal consultations	Identified limitations in existing tobacco and migration-related evidence, including limited attention to knowledge and attitudes, language accessibility, and heterogeneity within populations with migration backgrounds	Survey objective broadened from tobacco-use behaviour alone to a multilingual KAB instrument with multidimensional migration-related measures. The instrument was positioned as complementary to existing population-based surveillance rather than as a replacement.
Step 2: Developing the survey outline	Community representatives; practitioners working with migrant populations; individuals with migration backgrounds; researchers with migration backgrounds	Workshops, Group discussions; feedback rounds; individual exchanges; email comments	Assessed relevance and comprehensibility of proposed survey domains; identified topics considered important for diverse communities	Inclusion or strengthening of domains on language use, migration-related experiences, passive smoke exposure, social environment, emerging nicotine products and product-specific perceptions. Migration-related variables such as reasons for migration, main spoken language and integration-related variables were retained to support contextualised interpretation of tobacco and nicotine-related KAB.
Step 3: Survey design and item adaptation	Community representatives; Diaspora TV Switzerland; public health and social-sector practitioners; researchers; individuals with migration backgrounds	Item review; email feedback; face-to-face meetings; virtual meetings; phone calls; messaging applications; comments directly in SurveyMonkey	Reviewed item wording, response options, product categories, survey length, routing logic, visual aids and sensitivity of selected questions	The initial combined product-use list was restructured into individual product-specific question blocks, allowing each product to be introduced with a short description and visual aid. Product categories were expanded or refined to include cigarettes, heated tobacco products, e-cigarettes/vapes, waterpipe/shisha, cigars, cigarillos, pipe tobacco, snuff, snus with tobacco and nicotine pouches without tobacco. Cannabis and alcohol items were retained as contextual substance-use domains. Product descriptions were shortened to one or two plain-language sentences focused on product type, mode of use and identifiable characteristics, such as common brands. Highly detailed e-cigarette questions, including detailed questions on type, amount of liquid and nicotine concentration, were reduced or simplified to lower respondent burden.
Step 3: Translation, linguistic adaptation and pilot testing	Diaspora TV Switzerland, Native-speaking translators; cultural mediators; community representatives; people with migration backgrounds; research colleagues; pilot participants	Translation files; direct comments in Excel; email comments; SurveyMonkey comments; phone calls; face-to-face exchanges; online survey completion	Reviewed semantic equivalence, cultural appropriateness, natural phrasing, technical functioning and acceptability across language versions	Product images and descriptions were added to reduce ambiguity, especially for products that were difficult to distinguish across languages, such as e-cigarettes and heated tobacco products, or snus and nicotine pouches. Repeated statements and Likert-type response options were harmonised within and across language versions. Missing or inaccurate translations were corrected. Tamil and Serbian language versions were revised after feedback that initial translations felt artificial or insufficiently natural. Routing logic errors that prevented progression beyond background questions were corrected before field implementation.
Step 4: Implementation planning and dissemination strategy	Diaspora TV Switzerland; community organisations; migrant-support organisations; public health organisations; university and student networks; diplomatic and government-related actors	Partner mapping; dissemination planning; feedback on recruitment materials; community and organisational exchanges	Identified feasible recruitment channels and assessed accessibility of study communication materials	Multilingual flyers, website texts and video-based materials were developed to support community-based dissemination. Distribution was planned through organisational networks, social media, community events and academic settings, rather than relying only on conventional institutional recruitment channels.

### 2.3 Step 1: mapping of existing evidence and definition of survey objectives

In the first step, we mapped existing evidence on tobacco and nicotine product use among people with migration backgrounds. This included a global scoping review of studies on tobacco-related knowledge, attitudes and behaviours among people with migration experience, as well as a secondary analysis of Swiss Health Survey 2022 data (1, 17). These preparatory studies identified several methodological gaps, including a predominant focus on behavioural outcomes, limited measurement of knowledge and attitudes, restricted linguistic accessibility, and limited differentiation of migration-related experiences.

These findings informed the overall objective of the PATHS survey: to develop a multilingual instrument for assessing tobacco and nicotine-related knowledge, attitudes and behaviours among people with migration backgrounds in Switzerland, while capturing migration background as a multidimensional set of experiences rather than a single categorical indicator. The instrument was designed to complement existing population-based surveillance systems by generating more detailed explanatory evidence among populations that may be underrepresented in standard national-language surveys.

Participatory input at this stage contributed to refining the study focus by highlighting the relevance of migration-related experiences, language accessibility, and culturally shaped perceptions of tobacco and nicotine products.

### 2.4 Step 2: developing the survey outline

Based on the findings from Step 1, we developed a survey outline, defining key domains, relevant communities, and overall survey structure. The KAB framework guided the organisation of main domains, ensuring coverage of knowledge, attitudinal, and behavioural dimensions related to tobacco and nicotine products. Additional domains included sociodemographic characteristics, migration-related variables, social environment, health status, language use and exposure to tobacco-related environments.

Participatory input was used to assess whether the proposed domains were relevant, understandable and sufficiently comprehensive for the intended study population. Community and practice actors highlighted the importance of language barriers, migration-related experiences, product-specific concerns, passive smoke exposure and emerging tobacco and nicotine products. This input informed the inclusion of a wide range of product categories, including cigarettes, e-cigarettes, heated tobacco products, nicotine pouches and shisha.

### 2.5 Step 3: survey design, item adaptation, translation and pilot testing

#### 2.5.1 Item selection and adaptation

The PATHS survey was developed by integrating and extending questions from established national and international survey instruments. Rather than adopting any single instrument in full, we selected items according to their relevance for the PATHS objectives, their alignment with the KAB framework, and their suitability for multilingual administration among people with diverse migration-related experiences.

Items assessing tobacco and nicotine-related KAB were informed by the WHO Global Adult Tobacco Survey (WHO GATS), the Swiss Health Survey and the Health and Lifestyle Survey in Switzerland (45–47). These sources provided core items on tobacco use, perceived health risks, passive smoke exposure, cessation and attitudes towards tobacco and nicotine products. Items were adapted where necessary to reflect the contemporary product landscape in Switzerland, including e-cigarettes, heated tobacco products, nicotine pouches and shisha.

Socioeconomic, cultural, and religious background variables were informed by the Interdisciplinary Study of Inequalities in Smoking, including items on religious affiliation and participation, which have been shown to intersect with smoking-related norms and behaviours (22, 48, 49). Migration-related dimensions were informed by the International Organization for Migration’s individual factors assessment toolkit, which offered nuanced measures of migration background, as well as reasons for migration that were adapted to the Swiss context (50). These included country of birth, parental country of birth, nationality, year of arrival in Switzerland, residence status, reasons for migration, language use and national-language proficiency.

#### 2.5.2 Participatory-informed refinement of content and format

The draft questionnaire was refined through iterative feedback from community and public health actors, translators, researchers and people with migration backgrounds. We describe the process as participatory-informed and co-creation-oriented because participatory input shaped several core elements of the survey.

Input was collected through workshops, consultations, in-person group discussions, joint feedback sessions, email review rounds, comments in translation files, individual virtual meetings, phone calls, messaging applications and item-specific comments entered directly in the SurveyMonkey platform. Actors included a migrant-led multilingual media organisation (Diaspora TV Switzerland), community representatives, native-speaking translators, individual contacts from different linguistic and migration backgrounds, multilingual research colleagues with migration backgrounds, and health and social-sector actors working with migrant populations. Relevant actors were recruited largely through personal and professional networks, as well as through Diaspora TV Switzerland, which engages a wide network of community representatives.

Feedback informed both content and format. Content-related changes included the inclusion or refinement of items on shisha, passive smoke exposure, cannabis and alcohol use, product-specific risk perceptions and migration-related experiences. Format-related changes included simplification of wording, reduction of survey length, adjustment of routing logic, inclusion of visual aids showing tobacco and nicotine products, and refinement of communication materials. It also supported the choice of an online survey format using the SurveyMonkey Inc. platform, based on its accessibility and usability for diverse populations (51).

#### 2.5.3 Translation and linguistic validation

Linguistic and cultural accessibility was treated as a core design component of the PATHS survey. Languages were selected based on demographic data on major populations with a migration background in Switzerland and complemented by participatory prioritisation with community actors to identify language groups for whom national-language surveys may present access barriers (52, 53).

Translations were conducted in collaboration with Diaspora TV Switzerland and native-speaking translators or cultural mediators. Rather than treating translation as literal word substitution, the process focused on semantic equivalence, comprehensibility and cultural appropriateness.

Translation and adaptation took place iteratively. Since not all language versions were completed simultaneously, review and refinement occurred in parallel across languages. For instance, when comments were made in translation files, they were addressed in those files before upload to SurveyMonkey. When comments were made directly in the SurveyMonkey system, changes were implemented directly in the online instrument in order to efficiently address specific issues, while maintaining consistency across versions.

#### 2.5.4 Pilot testing and revision criteria

Pilot testing was conducted on a rolling basis for 4 months, before finalisation of the survey. Because language versions became available at different points in time, pilot testing was organised iteratively as each language version was finalised and uploaded to the online survey platform.

Each piloted language version was reviewed by approximately two to three individuals fluent in the respective language. Pilot testing was conducted for Albanian, English, Italian, Portuguese, Serbian, Spanish, Tamil, Turkish, and Ukrainian versions. Participants included people with migration backgrounds, community representatives, translators, research colleagues and general participants each fluent in their respective language. Several individuals occupied more than one of these roles (e.g., research colleague with a migration background and acting as a community representative).

The pilot was administered online using SurveyMonkey. Participants were asked to complete the survey and provide feedback on wording, comprehension, translation quality, response options, survey length, routing logic, technical functioning, product images and perceived sensitivity of selected items. Feedback was collected through several channels, that were most practical for the individual, including email comments, direct comments in translation files (Microsoft Excel), face-to-face discussions, phone calls, messaging applications (e.g., WhatsApp, iMessage) and item-specific comments entered directly in SurveyMonkey.

Feedback was reviewed by the research team and, where relevant, discussed with translators or community partners. Revisions were made when feedback indicated misunderstanding of an item, linguistically unnatural phrasing, technical problems with routing logic, excessive respondent burden, missing or unclear response options, inconsistencies between language versions, or potential sensitivity around specific wording. Similar to translation adaptations described prior, depending on the type of issue and the channel through which feedback was provided, revisions were made either in the translation files before upload or directly in the SurveyMonkey platform.

### 2.6 Step 4: implementation planning and dissemination strategy

Planning for survey implementation focused on maximising accessibility and reach. The survey was designed as an online instrument, allowing for flexible, snowball dissemination through community organisations, cultural associations, migrant support structures, social media, and academic networks. Rather than relying solely on conventional institutional recruitment channels, implementation planning emphasised collaboration with actors already embedded in the social and linguistic contexts of the communities we are hoping to reach.

We mapped potential partners representing communities reflected in the survey language versions, as well as organisations involved in migrant integration, public health, education and social support. Multilingual recruitment materials were developed in collaboration with community partners, including flyers, website texts and video-based materials. Follow-up measures were planned to support distribution through their partner networks and community events, university settings and social media platforms, such as Facebook, Instagram, Telegram, and YouTube.

## 3 Results

The final PATHS survey instrument consisted of a comprehensive, online, modular questionnaire on the SurveyMonkey Inc. platform, designed to capture tobacco and nicotine product use within a KAB framework and a multidimensional conceptualisation of migration background (51).

The questionnaire included the following core domains:

- Tobacco and nicotine product use (initiation, current use, cessation)
- Knowledge and perceptions of tobacco and nicotine products
- Attitudes towards smoking and nicotine product use
- Social environment and peer influences
- Sociodemographic characteristics and migration background
- Migration-related factors (e.g., mother tongue and national language fluency, integration-related variables, reasons for migration)
- Health status and well-being

In total, the questionnaire comprised a total of 136 items: 43 knowledge items, 20 attitude items, and 41 behaviour-related items, complemented by 32 sociodemographic and migration-related questions, with adaptive routing logic implemented to reduce respondent burden. The survey was developed and translated in 13 languages (Albanian, Arabic, English, French, German, Italian, Portuguese, Serbian, Spanish, Tamil, Tigrinya, Turkish, and Ukrainian). During pilot testing, the average completion time was 10–15 min, with a completion rate of approximately 80%, indicating good feasibility and acceptability across diverse participant groups.

A key feature of the instrument was the operationalisation of migration beyond a binary, categorical indicator. In addition to standard variables such as country of birth and parental country of birth, the survey captured a broader set of migration-related dimensions, including year of arrival in Switzerland, nationality at birth, residency status (including permit type and undocumented status), living situation (e.g., asylum centre), reasons for migration (e.g., education, employment, conflict or persecution), mobility history (number of countries lived in for more than 90 days), and linguistic factors such as main spoken language and fluency in Swiss national languages. Religious affiliation, religious practices, and perceived importance of religion were also included.

### 3.1 Instrument adaptations retained after participatory refinement

The participatory-informed development process resulted in several concrete adaptations that were retained in the final PATHS instrument. These adaptations are summarised across development phases in Table 2.

A central adaptation concerned the organisation of product-specific questions. In an earlier version, tobacco and nicotine products were presented in a combined multiple-choice item. Feedback from community actors indicated that this format was difficult to answer because several products were not consistently recognised or clearly distinguished, particularly newer or less familiar products such as heated tobacco products, e-cigarettes, snus and nicotine pouches. In the final instrument, these products were therefore separated into individual product-specific question blocks. This allowed each product to be introduced with a short description and visual aid before respondents answered product-specific knowledge, attitude or behaviour questions. In subsequent steps, further adaptation concerned wording and respondent burden. Earlier product descriptions were considered too technical or too lengthy for a broad community-based survey. They were therefore shortened to one or two plain-language sentences focusing on the type of product, how it is consumed and key identifiable characteristics, such as common brand examples where relevant. Similarly, highly detailed questions on e-cigarette type, amount of liquid consumed and nicotine concentration were reduced or simplified because they were considered too demanding for a general public audience.

The final instrument also incorporated visual aids with product images and image descriptions after feedback indicated uncertainty in distinguishing products across language versions. These visual supports were considered particularly helpful where community representatives reported that product terminology was unfamiliar or inconsistently used in their languages, such as with e-cigarettes and heated tobacco products, or snus and nicotine pouches.

As part of the participatory refinement process, pilot testing involved (*n* = 15) participants from diverse linguistic and migration backgrounds across 9 language versions. This phase confirmed the overall feasibility of the instrument and informed final adaptations. These included correcting routing logic that had prevented respondents from progressing beyond selected background questions, harmonising repeated statements and Likert-type response options across language versions, and correcting missing or inaccurate translations. Additional review of Tamil and Serbian language versions indicated that some translations felt artificial or insufficiently natural, leading to further refinement for fluency and comprehensibility.

### 3.2 Implementation readiness and dissemination outputs

**Figure 3:**
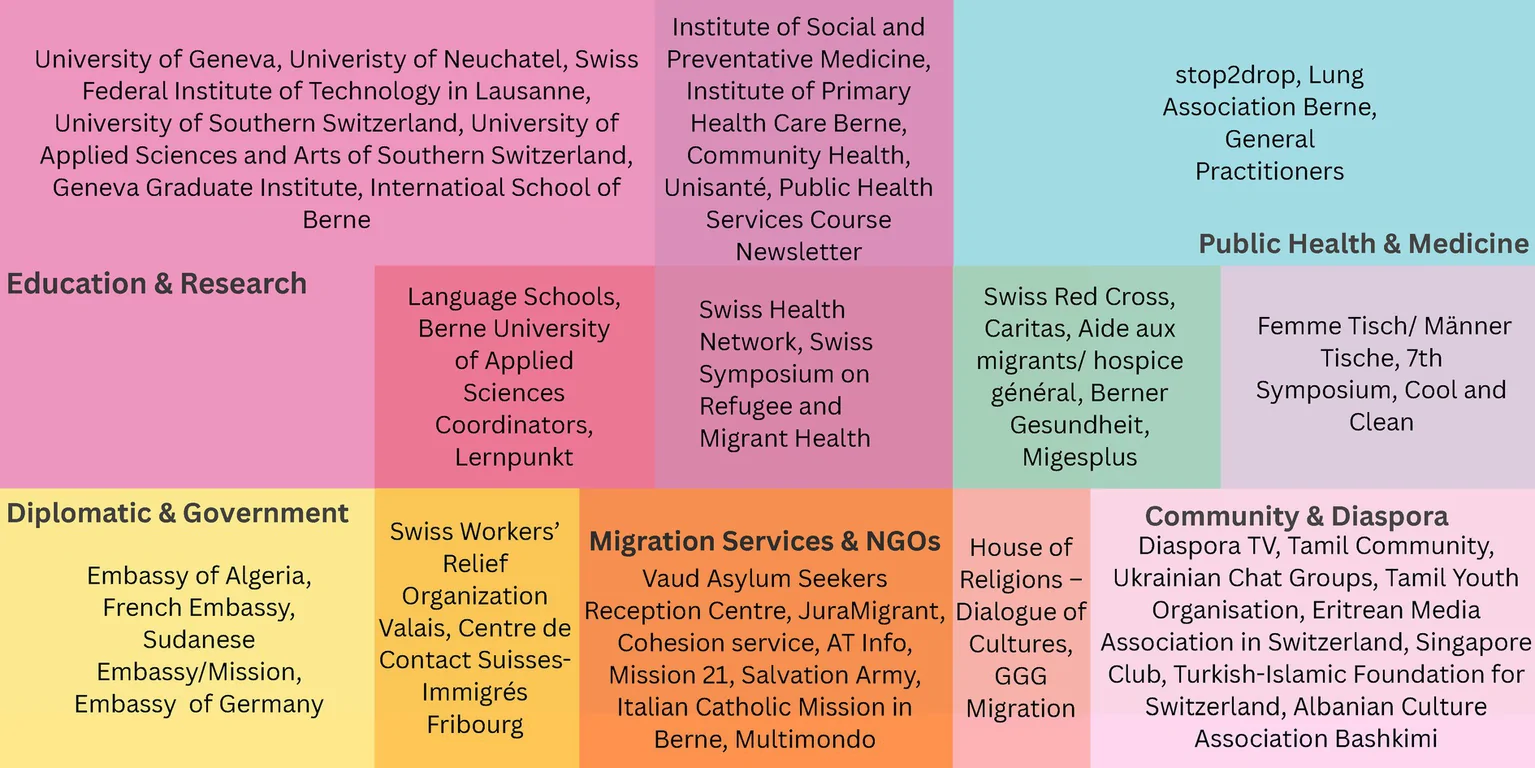
Thematic categorisation of potential distribution partners (Bern, Switzerland, 2026). The following names, such as Lernpunkt and Multimondo, are used in their original language, as they have no established English translation.

As part of the implementation planning process, we mapped a network of 53 potential partners. Potential partners represented communities reflected in the survey language versions, as well as institutions involved in migrant integration, such as community-based groups (*n* = 8), public health organisations (*n* = 4), university and student organisations (*n* = 7), diplomatic and government institutions (*n* = 4) and migration services and NGOs (*n* = 10). Additionally, the majority of institutions and organisations spanned more than one category (*n* = 20; see Figure 3).

Multilingual recruitment materials were developed in collaboration with Diaspora TV to support accessible and culturally adapted dissemination. These included three versions of survey flyers translated into 13 languages, a dedicated multilingual website providing study information and survey access, and video-based materials tailored to diverse linguistic audiences.

## 4 Discussion

This study presented the development of a multilingual, participatory survey instrument designed to assess tobacco and nicotine product-related KAB among people with migration backgrounds in Switzerland. Existing tobacco-related KAB studies have contributed important evidence on knowledge, attitudes and behaviours in specific populations and settings, but they often remain focused on single tobacco products, general population samples, or behaviour-oriented surveillance questions (1). For instance, the core questionnaire of the WHO GATS, seen as the global standard to systematically monitor tobacco use, only has five survey items on knowledge, attitudes and perceptions (45). Moreover, the WHO GATS largely omits question blocks on more recent products that are growing in popularity, such as heated tobacco products, and nicotine pouches. In Switzerland, population-level tools, including the Swiss Health Survey and the Health and Lifestyle Survey mirrored global methodological imbalances and has limited linguistic accessibility. The PATHS survey expands on the literature and existing KAB tools by integrating KAB domains of more tobacco and nicotine products, with a multi-lingual and migration-sensitive instrument structure.

A central contribution of this work thus lies in demonstrating how survey design can move beyond behaviour-dominant measurement towards a more comprehensive understanding of the determinants of tobacco and nicotine product use. It should not be understood that existing population-based surveys, such as the Swiss Health Survey, are weak or lack utility. They do provide valuable prevalence data, yet their ability to capture how knowledge, perceptions, and social context shape behaviour, particularly in diverse populations could be expanded by a complementary PATHS approach. It is by embedding additional KAB dimensions and integrating these within a migration-sensitive conceptualisation that PATHS enables a more integrated analysis of behavioural mechanisms. This is particularly relevant in tobacco prevention, where product use may be influenced not only by individual preferences, but also by risk perceptions, or social acceptability (23, 54).

Rather than treating migration as a static or binary characteristic, the PATHS instrument operationalises migration background as a multidimensional set of lived experiences, including migration history, legal status, language use, and social and cultural factors. This responds to critiques in migration and health research that broad labels such as “migrant” or “immigrant” versus “non-migrant,” or “native” can obscure heterogeneity and risk essentialising diverse populations when used without more contextual information (21, 55, 56). A multi-dimensional approach allows researchers to examine how different migration-related conditions intersect with tobacco and nicotine product-related KAB and may help to identify mechanisms underlying observed inequalities in tobacco and nicotine product use, while avoiding the interpretation of migration background itself as a direct causal explanation.

Beyond deciding which migration-related dimensions to measure, developing an instrument for culturally and linguistically diverse populations requires attention to whether questions retain accessible and sufficiently comparable meanings across contexts (57). Cross-cultural adaptation is therefore not reducible to literal translation; it also requires attention to conceptual and cultural equivalence, comprehensibility, response processes and pretesting. Cognitive testing among Latino immigrants, for example, found that general question-design defects and sociocultural interpretation problems were more common than literal translation problems (58). Research on co-design with culturally and linguistically diverse communities similarly identifies communication, trust, power differentials and differing understandings of health and services as recurrent challenges (11, 38). Broader cross-language research further suggests that language choice can shape the meaning and cultural nuance of participants’ responses rather than functioning only as a logistical consideration (19, 20).

The PATHS development process confirmed several question-design and interpretation challenges in a survey context. Some translations that were technically acceptable were perceived as unnatural or artificial by fluent speakers; repeated response categories were not rendered consistently across and within language versions; and the names of newer tobacco and nicotine products did not always support reliable recognition or differentiation. Detailed product descriptions and lengthy questions also increased cognitive burden. In response, the final instrument used product-specific question blocks, short plain-language descriptions, visual aids, harmonised response categories and iterative review by fluent community members (see Table 2). These adaptations illustrate the value of CASM-informed refinement, because the identified problems concerned comprehension, judgement and response mapping as well as language.

PATHS can also be situated more precisely within the range of participatory approaches described in migrant and public health research. Its early engagement of community and practice actors, iterative refinement, involvement of multilingual intermediaries and adaptation of both the questionnaire and dissemination materials are consistent with co-creation approaches that use experiential and practice-based knowledge to improve contextual fit (2, 10, 12, 34, 37, 38, 59). The approach also shares features with the Ophelia process applied with migrant communities, particularly the use of community knowledge to identify relevant needs and refine the accessibility of proposed solutions (40). However, PATHS did not apply an equivalent formal co-design protocol across the full research cycle, and PATHS differs from full community-based participatory research and more emancipatory models of participation (29). The project was researcher-initiated, shared governance was not established across all phases, and co-evaluation was not built into the complete development process. Participation therefore ranged primarily from consultation to partnership during survey design, translation, pilot testing and dissemination planning, rather than extending to community control (10, 39, 42, 60). Describing the approach as participatory-informed and co-creation-oriented is consequently more accurate than claiming full community-based participatory research. This positioning is consistent with migrant-health literature arguing that the appropriate form and level of participation should be tailored to the research context, while also identifying opportunities to deepen community involvement in future interpretation, dissemination and subsequent instrument refinement. These methodological gains were accompanied by tensions between contextual adaptation, standardisation and the resources required for sustained participation.

### 4.1 Trade offs

The development of a comprehensive and inclusive survey instrument entailed trade-offs. The integration of items from multiple validated instruments and the inclusion of additional domains increased the complexity and length of the survey, requiring careful balancing between comprehensiveness and respondent burden. While adaptive routing logic and participatory feedback helped mitigate these challenges, such approaches remain time and resource-intensive and may not be feasible in all research contexts. Furthermore, adaptations of validated items, while necessary for contextual relevance, may affect strict comparability with original instruments, although efforts were made to preserve core constructs wherever possible. As such, an additional key challenge included balancing academic rigor, and methodological integrity, while adapting the survey to community needs. This tension between cultural relevance and cross-version equivalence is well recognised in cross-cultural instrument development (57). Iterative review, simplified wording and visual aids helped mitigate these challenges, but multilingual adaptation and sustained stakeholder engagement required substantial time and resources (35, 36). Another challenge was navigating power dynamics with community representatives, especially regarding academic knowledge, or on the other hand access to communities. Participatory migrant-health research similarly cautions that fragmented community landscapes, unequal power relations and operational barriers may privilege organised, accessible or institutionally connected voices (61, 62). Fundamentally, sustaining interactions and collaborations required long-term investment beyond the initially planned research timeline. The PATHS study was supported by dedicated funding that enabled the allocation of resources to these activities.

### 4.2 Public health implications

In contexts characterised by linguistic diversity and heterogeneous populations, standardised survey instruments may fail to capture relevant dimensions of health behaviour. The combination of KAB frameworks, migration-sensitive measures, and participatory development offers a promising pathway to improve both the inclusiveness and explanatory power of survey-based research. While the specific design choices of PATHS are context-dependent, the underlying principles of early community engagement, iterative refinement, and multidimensional conceptualisation of key variables are transferable to other settings and health topics (63).

Rather than presenting PATHS as a universal model, this study positions it as one response to identified limitations in existing research. Its feasibility depends on available resources, research priorities, and the willingness to engage with more complex and time-intensive design processes. At the same time, the PATHS instrument demonstrates potential for broader application, including integration into population-based survey systems. Beyond its contribution to survey methodology, PATHS has implications for equity-oriented tobacco prevention (64). More inclusive and disaggregated evidence may support efforts to reduce health inequalities under Sustainable Development Goal 10, strengthen tobacco control under SDG target 3.a, and improve the availability of data differentiated by migratory status under SDG target 17.18 (65). The collaborative role of community, public health and migration-sector organisations also reflects the emphasis placed on multi-stakeholder and civil-society partnerships under SDG 17 (65). Organisations working with people with migration backgrounds thus play an important role, not only as recruitment partners, but also as knowledge intermediaries who can support interpretation, dissemination and translation of findings into practice.

In particular, the differentiated measurement of migration background, the integration of KAB, and the inclusion of a wide range of tobacco and nicotine products provide added analytical value compared to standard surveillance instruments, such as the Swiss Health Survey. These features enable more nuanced and context-sensitive analyses of health behaviours in diverse populations. More broadly, this work highlights the importance of recognising survey development as a substantive methodological phase, shaping not only how data are collected, but also the kinds of knowledge that can ultimately be produced. Future implementation will provide insights into the effectiveness of community-based dissemination strategies.

## Data Availability

The original contributions presented in the study are included in the article/supplementary material, further inquiries can be directed to the corresponding author.
